# Influence of Lipid Sources on Performance, Egg Quality, and Metabolism in Laying Quails

**DOI:** 10.3390/ani15213120

**Published:** 2025-10-27

**Authors:** Jean Kaique Valentim, Felipe Cardoso Serpa, Maria Fernanda de Castro Burbarelli, Alexander Alexandre de Almeida, Vivian Aparecida Rios de Castilho Heiss, Paulo Henrique Braz, Claudia Andrea Lima Cardoso, Claudia Marie Komiyama, Fabiana Ribeiro Caldara, Arele Arlindo Calderano, Sarah Sgavioli, Rodrigo Garofállo Garcia

**Affiliations:** 1 Department of Animal Production, Institute of Animal Science, Federal Rural University of Rio de Janeiro, Seropédica 23890-000, RJ, Brazil; jean.valentim@ufrrj.br(J.K.V.); 2 Faculty of Agricultural Sciences, University Federal da Grande Dourados, Dourados 79804-970, MS, Brazil; felipe.c.serpa@gmail.com (F.C.S.); mariaburbarelli@ufgd.edu.br (M.F.d.C.B.); alexanderalmzootec@gmail.com (A.A.d.A.); viviancastilho@live.com (V.A.R.d.C.H.); claudiakomiyama@ufgd.edu.br (C.M.K.); fabianacaldara@ufgd.edu.br (F.R.C.);; 3 College of Veterinary Sciences, Universidade Federal da Fronteira do Sul, Chapecó 89815-899, SC, Brazil; paulo.braz@uffs.edu.br (P.H.B.); 4 Course of Chemistry, University Estadual do Mato Grosso do Sul, Dourados 79804-970, MS, Brazil; claudiacardosouems1@gmail.com (C.A.L.C.); 5 Department of Animal Science, University Federal de Viçosa, Viçosa 36570-900, MG, Brazil; 6 Graduate Program in Animal Science (PPGCA), Pontifícia Universidade Católica do Paraná (PUCPR), Curitiba 80215-901, PR, Brazil

**Keywords:** fats, laying phase, metabolizable energy, oils, quail farming

## Abstract

**Simple Summary:**

This study investigated how different types of dietary fats affect the performance and egg quality of Japanese quail during the laying period. A total of 350 quail, 60 days old, were fed diets containing soybean oil, corn oil, canola oil, sunflower oil, or poultry fat. The birds were randomly divided into groups and their performance was measured every 28 days, while egg quality was evaluated at the end of each period. At 84 days, one bird from each group was analyzed to study nutrient use, blood characteristics, and organ health. The results showed that sunflower oil improved egg production, egg mass, and feed efficiency; poultry fat enhanced yolk color and other egg quality traits; and canola oil increased nutrient digestibility. None of the fat sources caused negative changes in blood parameters or organ traits. Overall, all diets maintained the birds’ normal physiological health. These findings suggest that different lipid sources can be used in quail diets according to their cost and availability, allowing producers to maintain good productivity and egg quality without affecting bird health.

**Abstract:**

Japanese quail production can be optimized by selecting appropriate dietary lipid sources, yet comparative effects on performance and egg quality during the laying phase are not fully established. This study evaluated the impact of five lipid sources, namely soybean oil, corn oil, canola oil, sunflower oil, and poultry fat, on performance, egg quality, nutrient metabolism, serum metabolites, and organ traits of 350 Japanese quail aged 60 days with an average weight of 170 ± 10 g. Birds were assigned to diets containing 2800 kcal/kg in a completely randomized design with 10 replicates of seven birds each. Performance was recorded over three 28-day periods and egg quality assessed at the end of each period; at 84 days, one bird per replicate was sampled for nutrient metabolism, serum metabolites, and organ characteristics, and a metabolism trial estimated metabolizability coefficients and metabolizable energy. Data were analyzed by Tukey’s test at the 5% level. Egg production (*p* = 0.010) and marketable egg production (*p* = 0.008) were highest with soybean, corn, and sunflower oils, while feed conversion per dozen eggs was less efficient with canola oil (*p* = 0.048). Egg quality differed in specific gravity (*p* = 0.027), yolk color (*p* = 0.008), Haugh unit (*p* = 0.011), and air cell size (*p* = 0.001), with poultry fat improving yolk color and Haugh unit. Canola oil increased dry matter (*p* = 0.027) and ether extract digestibility (*p* = 0.026), while serum metabolites, organ weights, and reproductive traits were not affected (*p* > 0.05). All diets supported physiological health, and lipid sources can be chosen according to cost and availability to optimize quail production without compromising performance or health.

## 1. Introduction

In practice, nutritionists frequently incorporate vegetable oils and/or animal fats into bird diets to achieve optimal energy levels. These lipids not only increase dietary energy density [1] but also provide essential nutrients that are fundamental for the proper functioning of the organism [2,3]. Their utilization is crucial in key physiological processes, including energy storage, maintaining cellular membrane integrity, and enhancing immunity [4]. Chemically, lipids consist of one, two, or three fatty acid molecules, classified as saturated when containing only single bonds between carbon atoms or unsaturated when containing one or more double bonds [5]. These structural differences, influenced by whether the lipid is of plant or animal origin, affect digestibility and, consequently, the efficiency of nutrient absorption and utilization in birds [6].

The inclusion of functional foods rich in fatty acids has become an important trend in precision nutrition due to their potential benefits, such as acting as natural protectors, stimulating cell renewal, reducing oxidative reactions, and promoting bird health and performance [7]. Amid growing concerns about sustainability and animal welfare, exploring alternative lipid sources offers opportunities to increase production while maintaining animal health. However, the high demand for oils and fats presents challenges for production, particularly regarding ingredient quality and consistency. Furthermore, scientific literature addressing specific nutritional parameters for quails is limited, making the formulation of optimized diets difficult. Consequently, alternative lipid sources such as palm, corn, and sunflower oils have been investigated, given their diverse nutritional profiles and potential to meet various energy and health requirements [8].

The inclusion of different lipid sources in laying quail diets is hypothesized to significantly influence nutrient metabolism, egg quality, and serum metabolites, with variations depending on whether the source is plant- or animal-based [9,10,11,12]. Compared to animal fats, plant-based lipids rich in unsaturated fatty acids are expected to enhance digestibility, improve egg quality, and support overall health [5]. Understanding these effects is essential for formulating efficient, cost-effective, and health-promoting diets, particularly in the context of precision nutrition and sustainable poultry production [10].

Therefore, this study aimed to evaluate the effects of different lipid sources in the diet of laying quails on nutrient metabolism, productive performance, egg quality, body temperature, and serum metabolites. The expectation is that analyzing these lipid sources will provide valuable data for developing more effective diets that meet the specific needs of quail, enhancing both productivity and animal welfare.

## 2. Materials and Methods

### 2.1. Animals and Housing

The experiment lasted 84 days and was divided into three periods of 28 days each. A completely randomized design was used with five treatments, corresponding to different lipid sources: soybean oil, corn oil, canola oil, sunflower oil, and poultry fat. The vegetable lipid sources were obtained from commercial oils, while the poultry fat was obtained from a feed factory that performs fat extraction. The diets were isoenergetic and isoproteic, containing 2800 kcal/kg, with 10 replications per treatment and seven Japanese quails (*Coturnix japonica*) per replicate, aged 60 days and weighing 170 ± 10 g at the peak of egg production, totaling 350 birds. The birds were housed in galvanized wire cages measuring 50 × 50 × 16.5 cm^3^ (length × width × height), each divided into two compartments of 25 × 50 cm^2^, totaling 1250 cm^2^. The stocking density was 178.5 cm^2^ per bird.

The experimental diets were provided ad libitum three times a day in galvanized metal trough feeders that extended throughout the entire length of the cages. The feeders were divided according to each treatment and replication. Water was also provided ad libitum to the nipple drinkers. The quails were provided with an artificial lighting program, maintaining 16 h of light per day using LED lamps.

The basal diet was composed of corn and soybean meal, without the inclusion of lipid sources, following the composition of the feedstuffs and the nutritional requirements established for the birds by Rostagno et al. [13], as shown in Table 1.

The nutritional composition of the experimental diets was analyzed for dry matter, gross energy, crude protein, calcium, phosphorus, and fatty acid content. Dry matter was determined gravimetrically by oven-drying at 105 °C until constant weight (rectilinear oven, model 315/3, Fanen^®^, Guarulhos, SP, Brazil). Gross energy was measured using a bomb calorimeter (model 6100, Parr^®^ Instrument Company, Moline, IL, USA). Total nitrogen was determined by the Kjeldahl method (INCT-CA N-001/1) using a nitrogen distiller (model TE-036/1, Tecnal^®^, Piracicaba, SP, Brazil) [14], and crude protein was calculated using a conversion factor of 6.25. Ash content was obtained by incinerating the samples in a muffle furnace at 600 °C (model F-3, Fornitec^®^, São Paulo, SP, Brazil). Calcium and phosphorus concentrations were determined using atomic absorption spectrophotometry (model AA-7000, Shimadzu^®^, Barueri, SP, Brazil) and UV/VIS spectrophotometry (model UV-5100, Tecnal^®^, Piracicaba, SP, Brazil), respectively, following Silva and Queiroz [15]. The analyzed values of gross energy, crude protein, calcium, and total phosphorus in the diet were 2850 kcal/kg, 19%, 3.00%, and 0.29%, respectively.

The daily management consisted of collecting and counting the eggs (daily counting of broken, cracked, soft-shelled, and shell-less eggs), providing the feed, cleaning the egg traps, and recording the maximum and minimum temperatures and relative humidity (RH).

The temperature and RH were monitored twice a day, at 8 am and 4 pm, via maximum and minimum thermometers (Instrutemp, ITHT-2250, São Paulo, Brazil) and dry and wet bulb thermometers (empresa, modelo, estado e país) positioned at the center of the shed at the height of the birds’ backs. The minimum temperature recorded was 20.1 ± 0.25 °C, and the maximum temperature was 35.9 ± 0.28 °C, with a maximum RH of 78.9 ± 1.8 (%) and a minimum of 49.8 ± 1.7 (%). Sixteen hours of light were provided daily throughout the experimental period. The lighting was controlled by an automatic timer. The activation of climate control systems and curtain control was based on daily temperature analysis.

### 2.2. Fatty Acid Content of the Diets

To identify the fatty acids in the diets, the rations were collected, homogenized, and frozen at −20 °C in a freezer to preserve the characteristics of the volatile fatty acids, and a representative sample was used for the analysis. The methodology used for lipid esterification followed the procedure described by Hulan et al. [16]. After obtaining the esters, they were analyzed using a Shimadzu GC-17 A gas chromatograph equipped with a flame ionization detector, manual injection, and a capillary column (CARBOWAX), with H_2_ used as the carrier gas. Calculations were performed through integration using a computer connected to the detector, extracting the total amount of fatty acids in the diets: 16:0 (Palmitic); 16:1 (Palmitoleic); 18:0 (Stearic); 18:1n − 9 (Oleic); 18:2n − 6 (Linoleic); 18:3n − 3 (Linolenic); 20:4n − 6 (Arachidonic); and 22:6n − 3 (Docosahexaenoic).

Each lipid source resulted in distinct fatty acid profiles, reflecting variations in the concentrations of saturated and unsaturated fatty acids. Diets supplemented with canola oil showed lower amounts of palmitic and stearic fatty acids, while poultry fat exhibited the lowest content of linoleic acid. Corn oil contributed to a higher amount of palmitic fatty acid, whereas sunflower oil presented the highest quantities of total fatty acids and linoleic acid (Table 2).

### 2.3. Performance

At the end of each experimental period, the remaining feed from each plot was weighed and subtracted from the amount of feed provided to obtain the feed intake. For birds that died during the period, their average intake was subtracted and corrected to obtain the average feed intake for the experimental unit.

The average egg production was determined by counting the number of eggs produced daily, including broken, cracked, and abnormal eggs (soft-shelled and shell-less eggs), expressed as a percentage relative to the average number of birds in the period (eggs/bird/day) and relative to the number of birds housed at the beginning of the experiment (eggs/bird housed).

To determine the production of marketable eggs, the number of broken, cracked, soft-shelled, and shell-less eggs was subtracted from the total egg production every 28 days. The ratio of intact eggs to total eggs produced during each period was then calculated.

All intact eggs produced in each replicate were weighed during the last three days of each 28-day period to obtain the average weight. The average egg weight was multiplied by the egg production per bird per day to obtain the total egg mass. The feed conversion per dozen eggs was calculated as the ratio of the total feed intake in kilograms divided by the number of dozens of eggs produced (kg/dozen), and the feed conversion per unit mass of eggs was calculated as the feed intake in kilograms divided by the total egg mass (kg/kg).

Bird mortality was monitored daily, and at the end of the experimental period, the bird viability rate was determined by calculating the difference between the number of live birds and the number of dead birds, expressed as a percentage.

### 2.4. Egg Quality

To quantify the components of the eggs, the weights of the yolk, albumen, and eggshell were evaluated. For this purpose, during the last three days of the experiment, three intact eggs from each replicate were collected, and three randomly selected eggs were chosen. The eggs from each replicate and each day were individually weighed on an analytical balance (model AL500C, Marte, Santa Rita do Sapucaí, Minas Gerais, Brazil) with a precision of 0.001 g. After weighing, the eggs were identified, and the following analyses were performed.

The eggs were placed in buckets with different concentrations of saline solution (NaCl), with densities ranging from 1.065 g to 1.100 g at intervals of 0.005 g. Using a hydrometer, eight buckets with saline solutions were set up, and the lowest to the highest concentrations of the eggs were evaluated for gravity, according to the methodology proposed by Castelló et al. [17].

After cracking, the eggshells, yolks, and albumen were separated on a flat surface, and yolk color was assessed using a portable colorimeter (Konica Minolta, CR 410, USA). The parameters measured included lightness (L), redness (a), and yellowness (b*) at three distinct points on the yolk surface, with the mean value calculated across these points. Eggshell color was also determined using a La Roche color fan on a 1–14 scale [18].

The heights of the yolk and albumen, as well as the diameter of the yolk, were measured via a caliper (Digimess, 110-284, São Paulo, Brazil) with the assistance of a tripod. The height of the yolk was measured in the central region, and the height of the albumen was measured 4 cm away from the yolk.

The yolk was separated from the albumen and weighed individually on a digital scale (model AL500C, Marte, Santa Rita do Sapucaí, Minas Gerais, Brazil). The weight of the albumen was obtained by subtracting the weight of the shell from the difference between the weight of the egg and the weight of the yolk. The weight of the shell was obtained after washing and drying in an oven at 65 °C for 72 h. The percentages of shell, yolk, and albumen were calculated by dividing these components by the weight of the egg and multiplying the result by 100.

After the shells were washed and dried, the shell thickness was measured at three different points via a precision micrometer with a resolution of 0.001 mm (Digimess, 110-284, São Paulo, Brazil), and the average of these three thickness points was calculated. The size of the air cell was measured after three eggs were cracked per experimental replicate via a precision micrometer with a resolution of 0.001 mm (Digimess, 110-284, São Paulo, Brazil).

The Haugh unit is a mathematical equation described by Eisen et al. [19], that correlates egg weight with the height of dense albumen.

HU = 100 log (H + 7.57 − 1.7 W^^0.37^)

H = height of the dense albumen (mm), and W = egg weight (g).

The yolk index was calculated as the ratio between the height and the diameter of the yolk, according to Moraleco et al. [20]. The yolk and albumen were mixed, and a sample portion was placed in the compartment of the Aqualab water activity analyzer (Decagon Devices Inc., Pullman, WA, USA), which uses the dew point principle according to the methodology of Scatolini-Silva et al. [21].

To determine the pH of the yolk and albumen, they were separated, placed in collecting flasks, and gently homogenized for measurement via a digital pH meter (Testo^®^ 205, São Paulo, Brazil), according to Souza et al. [22].

### 2.5. Nutrient Metabolism and Apparent Metabolizable Energy Corrected for Nitrogen Values

The coefficient of nutrient metabolism was determined via the total collection of excreta method with the use of an iron oxide marker at the end of the 84th evaluation day [23]. Collections were carried out twice a day, at 8:00 AM and 5:00 PM, for five days. All the cages were equipped with a preprepared tray for excreta collection.

The excreta were collected, placed in plastic bags, labeled by replication, and stored in a freezer at −16 °C. At the end of the experimental period, the amount of feed consumed and the total amount of excreta produced were determined. The excreta were thawed and homogenized, and a representative sample of approximately 200 g from each replicate was taken, weighed, and then placed in a forced ventilation oven at 55 °C for 72 h. The samples were subsequently exposed to air to reach equilibrium at ambient temperature and humidity.

They were then weighed, ground in a Willey mill with a 1 mm knife, and stored in a freezer at −20 °C for laboratory analysis of dry matter (DM), mineral matter (MM), crude protein (CP), and ether extract (EE). Apparent metabolizable energy (AME), nitrogen-corrected apparent metabolizable energy (AMEn), and metabolizability coefficient (MC) values were calculated via the equations proposed by Matterson et al. [24].

$$ME\;of\;TD\;or\;RD=\left(\frac{kcal}{kg}\right)=\left(\frac{GE\;ingested-GE\;Excreted}{Feed\;intake}\right)$$

$$\frac{kcal}{kg}=\left(\frac{GE\;ingested-GE\;Excreted}{Feed\;intake}\right)$$

$$AME\;of\;lipif\;source(\frac{kcal}{kg})=AME\;RD+\left(\left(\frac{AME\;TD-AME\;RD}{\%\;of\;replacement}\right)\right)$$

$$AME\;of\;TD\;or\;RD\left(\frac{kcal}{kg}\right)=\left(\frac{GE\;ingested-\left(GE\;excreted+8.22\times NB\right)}{Feed\;intake}\right)$$

$$AMEn\;of\;lipid\;source\left(\frac{kcal}{kg}\right)=\left(\frac{AMEn\;RD+\left((AMEn\;TD-AMEn\;RD\right)}{\%\;of\;replacement}\right)$$

where

TD = test diet;

RD = reference diet;

GE = gross energy and

NB = nitrogen balance = N ingested − N excreted.

The calculation of the metabolizability coefficient (MC) values from the total collection of excreta was performed using the following equation:

$$MC\left(\%\right)=\left(\frac{Amount\;of\;nutrient\;in\;the\;feed-mount\;of\;nutrient\;excreted}{Amount\;of\;nutrient\;in\;the\;feed}\right)\times 100$$

The metabolizable energy values of the lipid sources were obtained through a metabolism assay, resulting in the following values: the MEAn of soybean oil, corn oil, canola oil, sunflower oil and poultry fat was 8790.00 kcal/kg, 8773.00 kcal/kg, 8784.00 kcal/kg, 8788.00 kcal/kg, and 8681.00 kcal/kg, respectively. The MC (%) values for soybean oil, corn oil, canola oil, sunflower oil and poultry fat were 93.88%, 93.53%, 93.32%, 93.74% and 93.06%, respectively.

### 2.6. Serum Metabolites

The serum metabolites analyses were conducted at the veterinary medicine department of the Federal University of Fronteira do Sul—Realeza campus, PR. Blood samples were collected via cardiac puncture for blood analysis via 3 mL syringes with 25 × 0.8 mm^2^ needles without anticoagulants. The birds were placed in dorsal recumbency, and then the needle was inserted along the ventral floor of the thoracic inlet into the heart. The samples were immediately centrifuged to separate the serum and then frozen at −20 °C until the time of biochemical analysis. Cholesterol, triglycerides, AST, ALT, and glucose were evaluated. Blood samples were collected in plain tubes for serum separation (BD Vacutainer^®^, Becton, Dickinson and Company, Franklin Lakes, NJ, USA) were determined using commercial enzymatic colorimetric kits (Gold Analisa^®^ Gold Analisa Diagnóstica Ltd.a., Belo Horizonte, Brazil). Biochemical tests were performed via a spectrometer (BioPlus 200, Biotecnica Instruments, São Paulo, Brazil) according to the manufacturer’s instructions for the commercial kits. For lipid profile analysis (cholesterol and triglycerides), blood samples without anticoagulant were used, incubated in plastic vials for 1 h at room temperature, and then centrifuged at 3500 rpm and 4 °C for 15 min to separate the serum.

The measurements were obtained via commercial kits, and the samples were pre-pared and analyzed according to the manufacturer’s specifications. Three readings were taken using a spectrophotometer (Beckman Coulter, Brea, CA, USA; DU-800) at a wavelength of 500 nm.

### 2.7. Organ Characteristics

Slaughter and sample collection were performed at the end of the 84th day of the experiment. During this period, the birds underwent an 8 h fast, with no access to feed but water available ad libitum, and then one bird from each plot was selected, weighed, and identified with body weight within ±10% of the average weight of the experimental unit, totaling 50 slaughtered birds.

The birds were rendered unconscious by cervical dislocation, and manual bleeding was performed by cutting the jugular vein. After gentle scaling at 56 °C for 2 min, the birds were manually defeathered. The carcass was weighed, and the edible viscera (liver, gizzard, and heart) and inedible viscera (proventriculus, small intestine—duodenum, jejunum, and ileum—ceca, ovary, and oviduct) were removed.

All the viscera were weighed via a semianalytical balance (marca, modelo, estado e país). To measure the gizzard weight, the contents of the organs were removed while preserving the keratin that surrounds them. The inedible cuts (proventriculus, duodenum, jejunum, ileum, and cecum) were lightly compressed to remove the interior contents, and the clean tissue was weighed using a balance with a precision of 0.5 g (marca, modelo, estado e país). The relative weights of the edible and inedible viscera were calculated by dividing the absolute weight of the organ by the carcass weight and multiplying by 100.

### 2.8. Digestive and Reproductive Tract Biometry

After each tissue (duodenum, jejunum, jejunum + ileum, ceca, ovary, and oviduct) was identified, the contents of the intestines were emptied. A 90 cm tape measure (with a precision of 0.1 mm) was used to measure all segments. To obtain the relative length values, the measurements of each segment were divided by the total length of the organ and multiplied by 100.

### 2.9. Statistical Analysis

Data on performance and egg quality were collected at the end of each 28-day period. The remaining data were collected at the end of the total experimental period (84 days). Regarding performance data, each group of seven birds was considered as one experimental unit, with ten replications. For egg quality data, each group of nine eggs (three eggs collected on three consecutive days) was considered as one experimental unit, with ten replications. For nutrient metabolism and apparent metabolizable energy corrected for nitrogen (AMEn) evaluations, at 84 days, each group of seven birds was considered as one experimental unit, with ten replications. For serum metabolites and organ characteristics evaluations, each bird was considered as one experimental unit, with a total of ten replications.

The data were subjected to tests for normality of residuals and homogeneity of variances using the Shapiro–Wilk and Levene’s tests, respectively. Data that did not meet these assumptions were transformed. An analysis of variance (ANOVA) was performed using the MIXED procedure of SAS 9.3 (SAS Institute, Cary, NC, USA, 2012). The statistical model included the random effect of replication within treatment, to account for random variation among replicates, and the fixed effect of period as a covariate. Treatment means for the evaluated variables were compared using Tukey’s test, also performed under the MIXED procedure. For all analyses, a 5% significance level was adopted.

## 3. Results

The inclusion of different lipid sources in the diets of laying Japanese quail significantly influenced performance and egg quality traits (Table 3 and Table 4). Egg production (*p* = 0.010) and marketable egg production (*p* = 0.008) were higher in birds fed soybean, corn, and sunflower oils, without differing from poultry fat. Feed conversion per dozen eggs were less efficient (*p* = 0.048) in the canola oil group (1.71), while the other lipid sources showed similar conversion values (1.63 to 1.64) (Table 3).

Regarding egg mass (*p* = 0.008), quail fed sunflower oil produced the highest egg mass (10.83 kg), whereas those fed canola oil exhibited the lowest (9.97 kg). Feed intake, feed conversion per egg mass and viability remained stable across all treatments (*p* > 0.05) (Table 3).

The egg quality parameters (Table 4) evaluated, including egg weight, yolk weight, shell weight, albumen weight, yolk color assessed using the Roche color fan, L and a values, albumen height (mm), yolk height (mm), yolk diameter (mm), shell thickness (cm), yolk index, percentages of yolk, shell, and albumen, albumen pH, yolk pH, and water activity, did not differ significantly among the evaluated groups (*p* > 0.05).

Egg quality characteristics revealed significant differences in specific gravity (*p* = 0.027), yolk color (*p* = 0.008), Haugh unit (*p* = 0.011), and air cell size (*p* = 0.001) (Table 4). Eggs from quail fed corn oil exhibited the highest specific gravity (1.074), not differing from the other treatments except for canola oil, which showed the lowest value (1.070). Yolk color, measured by the colorimetric parameter “b,” was most enhanced in diets containing poultry fat (37.50), not differing from the other treatments except for soybean oil, which presented the lowest value (35.79) (Table 4).

Haugh unit values, indicating albumen quality, were highest in eggs from quail fed poultry fat (89.64), not differing from soybean and sunflower oils, and lowest in those fed corn oil (86.91) and canola oil (87.19). Air cell size was smallest in eggs from quail fed sunflower oil (1.42 mm), not differing from soybean and canola oils, and largest in those fed poultry fat (2.30 mm) (Table 4)

Digestibility analysis revealed significant differences among lipid sources for dry matter (*p* = 0.027), ether extract (*p* = 0.026), and mineral matter (*p* = 0.001), as shown in Table 5. Diets containing canola oil exhibited the highest digestibility values for dry matter (84.65%) and ether extract (87.23%), whereas diets with poultry fat and corn oil showed the lowest digestibility for dry matter (79.38%) and ether extract (77.10%), respectively. The highest mineral matter digestibility was observed in quail fed poultry fat (66.55%), not differing from those fed soybean oil (63.87%) and sunflower oil (65.25%). Metabolizable energy (AMEn) and crude protein digestibility were not significantly affected by dietary lipid sources (*p* > 0.05) (Table 5).

No significant differences (*p* > 0.05) were observed for blood serum biochemical parameters, including AST, ALT, cholesterol, glucose, and triglycerides, remained stable across all lipid treatments (Table 6).

The absolute and relative weights of the liver, heart, gizzard, and proventriculus, as well as the oviduct, ovary, and intestinal segments (duodenum, jejunum+ileum, and cecum), are presented in Table 7 and no significant differences were observed among treatments (*p* > 0.05).

## 4. Discussion

The high concentrations of oleic acid in canola oil and of linoleic and linolenic acids in sunflower oil, as observed in the fatty acid composition analysis of the diets, may influence the metabolism and physiology of the birds [2]. The distinct fatty acid profiles indicate that the choice of lipid source can markedly affect the dietary balance of saturated and unsaturated fatty acids [10]. These differences underscore the importance of selecting appropriate lipid sources to achieve specific nutritional objectives and to optimize the metabolic health of quails. According to several authors [19,20,21], the inclusion of lipid sources in poultry diets is essential due to their role in host defense mechanisms and the presence of bioactive compounds with antioxidant properties, such as vitamins A and E, particularly abundant in canola and sunflower oils. These antioxidant substances neutralize oxidative stress generated during physical exertion, injuries, disease, and allergen exposure, thereby preventing oxidation of phospholipids and proteins essential to cell membrane integrity [22].

The results of the present study demonstrate that dietary lipid composition has a significant effect on the productive performance and egg quality of laying Japanese quails. Essential fatty acids, particularly linoleic acid, play a crucial role in physiological processes [17]. As a fundamental structural component of all cell membranes, linoleic acid directly influences their fluidity, flexibility, and permeability vital functions for nutrient transport and overall cellular health that support egg production [23]. Moreover, linoleic acid is a precursor of potent metabolic regulators known as eicosanoids (e.g., prostaglandins), which act as “local hormones” to modulate immune function and reproductive processes [25]. A deficiency in linoleic acid has been shown to negatively affect egg production and egg weight. These effects may explain the lower egg mass observed in birds fed canola oil, as this lipid source, although rich in monounsaturated oleic acid, contains a lower proportion of linoleic acid compared to the other tested sources [26].

Yolk color, as indicated by the “B” parameter, was improved in diets containing poultry fat due to the presence of xanthophylls such as lutein and zeaxanthin, which are known to enhance yolk pigmentation and, consequently, consumer preference [20]. Variations in Haugh unit values among lipid sources reflect differences in albumen protein stability [27]. Distinct metabolic pathways and energy efficiency associated with different fatty acid profiles may influence the allocation of resources for protein synthesis or preservation [28]. Poultry fat, with its more saturated and mixed fatty acid profile, is metabolized differently from highly unsaturated vegetable oils, which could have downstream effects on albumen protein synthesis, potentially explaining the higher Haugh unit values observed in poultry fat diets [29].

Air cell size was smaller in eggs from quails fed sunflower oil and larger in those fed poultry fat, correlating with moisture loss through the shell [27]. The fatty acid profile of sunflower oil, particularly rich in linoleic acid [30], may contribute to an improved shell membrane structure that limits water vapor diffusion [31]. Diets high in linoleic acid lead to its incorporation into membrane lipids, enhancing structural integrity and hydrophobic properties. This creates a more effective barrier against moisture loss, resulting in a smaller air cell. In contrast, the fatty acid profile of poultry fat, containing less linoleic acid, may result in a membrane structure less effective at retaining moisture.

The higher digestibility of dry matter and ether extract observed in diets with canola oil can be attributed to its unsaturated fatty acid profile, which facilitates lipid emulsification and absorption in the gastrointestinal tract [29]. Unsaturated fatty acids, predominant in canola oil, are utilized more efficiently than saturated fatty acids, leading to higher metabolizable energy values [32]. Additionally, oil supplementation can reduce digesta passage rate, allowing more time for nutrient absorption [33]. The rapid gastrointestinal transit times in quails, as described by Göçmen et al. [34], highlight the importance of highly digestible lipid sources for optimal nutrient absorption, making the fatty acid profile of canola oil particularly advantageous for this species.

The serum AST and ALT levels observed in this study were higher than the basal values reported in the literature for clinically healthy laying quails (AST ~ 121 U/L; ALT ~ 10 U/L), which can be attributed to the characteristic metabolic profile of laying birds, dominated by vitellogenesis [34,35]. This physiological process induces hyperproteinemia and hyperlipidemia to support egg formation, with total protein ranging from 3.7 to 6.6 g/dL and total cholesterol between approximately 105 and 200 mg/dL. The hormonal changes associated with the laying phase result in increased concentrations of total protein, albumin, cholesterol, and triglycerides [36,37].

Overall, these results highlight the importance of appropriately selecting lipid sources to optimize both productivity and egg quality in quail diets. The canola oil, enhanced nutrient digestibility, indicating your potential as a substitute for soybean oil, currently the most widely used lipid source in quail feeding [8]. The distinct fatty acid profiles of the evaluated lipid sources emphasize their potential physiological effects in Japanese quails [38]. In particular, the high oleic acid content of canola oil and the abundant linoleic acid in sunflower oil are noteworthy, as these fatty acids are essential for maintaining cell membrane fluidity, supporting efficient nutrient transport, and modulating immune function [39].

The superior digestibility of canola oil, likely due to its high unsaturated fatty acid content, enhances lipid emulsification and absorption [40,41], processes that are critical for energy metabolism and tissue repair. Conversely, the elevated linoleic acid concentration in sunflower oil, an essential fatty acid, may improve reproductive performance and modulate inflammatory responses [42,43], thereby contributing to superior egg production and mass. Collectively, these findings underscore the importance of targeted lipid supplementation in optimizing not only productive performance but also physiological health, particularly by modulating metabolic processes and maintaining cellular functional integrity.

## 5. Conclusions

The inclusion of different lipid sources in diets for laying Japanese quails demonstrated that all treatments were nutritionally adequate to maintain physiological health and egg production. Poultry fat improved egg quality, and canola oil promoted superior nutrient digestibility. These findings suggest that dietary lipid sources can be strategically adjusted based on availability and cost to optimize quail production without compromising performance or health.

## Figures and Tables

**Table 1:** Percentage and calculated composition of experimental diets in the production phase.

	Lipid Sources				
Ingredients	Soybean	Corn	Canola	Sunflower	Poultry Fat
Corn meal	56.291	56.291	56.291	56.291	56.291
Soybean meal 45%	30.707	30.707	30.707	30.707	30.707
Inert	1.059	0.963	1.023	0.816	0.888
Lipid source	2.674	2.770	2.710	2.917	2.845
Limestone	6.950	6.950	6.950	6.950	6.950
Dicalcium phosphate	1.026	1.026	1.026	1.026	1.026
Salt	0.343	0.343	0.343	0.343	0.343
DL-methionine, 98%	0.417	0.417	0.417	0.417	0.417
L-Lysine HCL, 76%	0.323	0.323	0.323	0.323	0.323
^1^ Vitamin Premix	0.100	0.100	0.100	0.100	0.100
^2^ Mineral Premix	0.100	0.100	0.100	0.100	0.100
Calculated nutritional composition					
Metabolizable energy (kcal/kg)	2800	2800	2800	2800	2800
Crude protein (%)	18.92	18.92	18.92	18.92	18.92
Digestible lysine (%)	1.15	1.15	1.15	1.15	1.15
Methionine + Cyst. (%)	0.942	0.942	0.942	0.942	0.942
Tryptophan digs. (%)	0.186	0.186	0.186	0.186	0.186
Threonine digs. (%)	0.733	0.733	0.733	0.733	0.733
Calcium (%)	3.0	3.0	3.0	3.0	3.0
Phosphorus available (%)	0.282	0.282	0.282	0.282	0.282
Sodium (%)	0.147	0.147	0.147	0.147	0.147

^1^ Vitamin premix/kg of diet. Folic acid (Min.) 145.4 mg. Pantothenic acid (Min.) 5931.6 mg. Choline (Min.) 121.8 g; niacin (Min.) 12.9 g; selenium (Min.) 480.0 mg. Vitamin A (Min.) 5,000,000.00 IU. Vitamin B12 (Min.) 6500.0 mcg. The vitamin B2 (Min.) 2000.0 mg. The vitamin B6 (Min.) 250.0 mg. Vitamin D3 (Min.) 1,850,000.00 IU. Vitamin E (Min.) 4500.0 IU. Vitamin K3 (Min.) 918.0 mg. ^2^ Mineral premix/kg. Copper (Min.) 7000.0 mg; Iron (Min.) 50.0 g. Iodine (Min.) 1500.0 mg. The manganese (Min.) content was 67.5 g, and the zinc (Min.) content was 45.6 g.

**Table 2:** Descriptive analysis of the fatty acid content of Japanese quail diets with different lipid sources included (% of total fatty acids).

Fatty Acid Profile	Soybean	Corn	Canola	Sunflower	Poultry Fat
Total Acids Fatty Ration	95.47	95.44	95.28	96.96	96.14
Palmitic (16:0)	13.95	16.37	8.95	11.95	20.56
Palmitoleic (16:1)	0.53	0.59	0.67	0.53	6.94
Stearic (18:0)	7.13	5.97	4.13	6.13	8.03
Oleic (18:1n − 9)	26.67	34.63	55.81	26.78	42.54
Linoleic (18:2n − 6)	44.82	36.51	24.35	49.32	16.97
Linolenic (18:3n − 3)	2.12	1.12	1.12	2.03	0.89
Arachidonic (20:4n − 6)	0.13	0.13	0.13	0.11	0.10
Docosahexaenoic (22:6n − 3)	0.12	0.12	0.12	0.11	0.11

Descriptive data analysis.

**Table 3:** Performance of Japanese quails in the laying phase fed diets supplemented with different lipid sources.

Variables	Lipid Sources					SEM	*p* Value
	Soybean	Corn	Canola	Sunflower	Poultry Fat		
Consumption/bird/day (g)	25.55	25.47	25.56	25.86	25.37	0.135	0.810
Eggs production (%)	94.52 A	94.84 A	90.65 B	95.17 A	93.95 AB	0.465	0.010
Marketable eggs production (%)	93.24 A	93.59 A	88.71 B	93.11 A	91.88 AB	0.459	0.008
Feed conversion/dozen (kg/dz)	1.63 B	1.63 B	1.71 A	1.63 B	1.64 B	0.011	0.048
Mass eggs (kg)	10.59 AB	10.60 AB	9.97 B	10.83 A	10.26 AB	0.089	0.008
Feed conversion/mass (kg/kg)	2.43	2.41	2.56	2.39	2.48	0.020	0.063
Viability (%)	99.85	99.85	99.67	99.91	99.53	0.098	0.704

A, B: Means followed by different letters on the line differ statistically at the 5% level of probability according to the Tukey test. SEM: Standard error of the mean.

**Table 4:** Egg quality of Japanese quails in the laying phase fed diets supplemented with different lipid sources.

Variables	Lipid Sources					SEM	*p* Value
	Soybean	Corn	Canola	Sunflower	Poultry Fat		
Egg weight (g)	11.220	11.140	11.020	11.220	11.930	0.050	0.256
Yolk weight (g)	3.640	3.610	3.560	3.600	3.460	0.024	0.130
Shell weight (g)	0.910	0.910	0.880	0.890	0.890	0.005	0.242
Albumen weight (g)	6.640	6.610	6.500	6.610	6.560	0.054	0.897
Gravity	1.072 AB	1.074 A	1.070 B	1.071 AB	1.073 AB	0.001	0.027
Fan	4.780	4.930	4.680	4.860	4.860	0.082	0.746
L	55.560	55.780	55.570	55.450	56.040	0.128	0.592
a	−1.940	−1.810	−1.980	−1.770	−1.630	0.044	0.070
b	35.79 B	36.95 AB	36.35 AB	36.20 AB	37.50 A	0.163	0.008
Albumen height (mm)	4.110	4.000	4.080	4.150	4.390	0.046	0.098
Yolk height (mm)	10.480	10.630	10.600	10.470	10.640	0.047	0.654
Yolk diameter (mm)	23.390	23.260	23.400	23.300	23.150	0.095	0.794
Shell thickness (cm)	0.210	0.210	0.200	0.200	0.200	0.001	0.265
Haugh unit	88.83 AB	86.91 B	87.19 B	87.83 AB	89.64 A	0.264	0.011
Yolk index	0.440	0.450	0.450	0.440	0.460	0.002	0.378
Yolk (%)	32.940	32.580	32.870	33.150	32.030	0.274	0.698
Shell (%)	8.160	8.220	8.080	7.980	8.200	0.038	0.253
Albumen (%)	58.910	59.180	58.980	58.850	59.790	0.289	0.755
Air chamber (mm)	1.75 BC	1.94 AB	1.81 BC	1.42 C	2.30 A	0.067	0.001
pH albumen	8.764	8.822	8.878	8.880	8.820	0.018	0.263
Yolk pH	6.360	6.431	6.402	6.465	6.543	0.025	0.196
Water activity	0.985	0.990	0.987	0.989	0.982	0.001	0.291

A–C: Means followed by different letters on the line differ statistically at the 5% level of probability. SEM: Standard error of the mean.

**Table 5:** Metabolizability coefficients of dry matter, ether extract, crude protein, and mineral matter and apparent metabolizable energy corrected for nitrogen of diets containing different lipid sources.

Variables	Lipid Sources					SEM	*p*-Value
	Soybean	Corn	Canola	Sunflower	Poultry Fat		
Dry matter (%)	84.13 AB	80.99 AB	84.65 A	82.21 AB	79.38 B	0.604	0.027
EE (%)	79.40 AB	77.10 B	87.23 A	84.66 AB	80.69 AB	1.150	0.026
CP (%)	71.15	67.58	69.41	67.80	66.30	0.978	0.587
M. Mineral (%)	63.87 AB	57.25 C	61.44 BC	65.25 AB	66.55 A	0.675	0.001
AMEn (kcal/kg)	2.818	2.747	2.746	2.751	2.752	2.401	0.890

EE (%): Ether extract. CP (%): Crude protein. M. Mineral: mineral matter. AMEn: Apparent metabolizable energy corrected for nitrogen. A–C: Means followed by different letters on the line differ statistically at the 5% probability level according to Tukey’s test. SEM: Standard error of the mean.

**Table 6:** Biochemical analyses of the blood serum of Japanese quails in the laying phase fed diets supplemented with different lipid sources.

Variables	Lipid Sources					SEM	*p*-Value
	Soybean	Corn	Canola	Sunflower	Poultry Fat		
AST (UI/L)	291.24	362.78	257.43	328.76	326.30	32.18	0.201
ALT (UI/L)	2.890	2.978	2.204	2.345	2.968	0.782	0.327
Cholesterol (mg/dL)	109.70	95.70	98.40	103.90	110.00	11.32	0.858
Glucose (mg/dL)	266.60	257.60	260.70	268.60	261.00	9.22	0.912
Triglycerides (mg/dL)	315.20	329.30	294.70	398.80	358.60	51.86	0.656

SEM: Standard error of the mean. ALT: alanine aminotransferase. AST: aspartate aminotransferase (AST).

**Table 7:** Absolute and relative weights of organs and biometrics of the reproductive and gastrointestinal tracts of Japanese quails in the laying phase fed diets supplemented with different lipid sources.

Variables	Lipid Sources					SEM	*p*-Value
	Soybean	Corn	Canola	Sunflower	Poultry Fat		
Liver (%)	5.91	5.24	5.38	5.43	5.15	0.138	0.484
Heart (%)	1.64	1.44	1.49	1.60	1.58	0.028	0.143
Gizzard (%)	4.44	4.43	4.81	4.61	4.29	0.095	0.896
Proventriculus (%)	0.73	0.87	0.81	0.82	0.74	0.171	0.055
Oviduct (%)	51.98	56.84	55.13	52.81	54.37	1.187	0.738
Ovary (%)	47.68	43.15	43.86	49.41	45.63	1.214	0.457
Duodenum (%)	10.22	10.88	10.96	11.01	9.54	0.636	0.535
Jejunum+Ileum (%)	43.60	45.51	46.84	45.00	44.02	0.670	0.586
Cecum (%)	27.58	27.40	23.35	27.17	28.40	0.757	0.241
Duodenum (% cm)	18.29	18.66	18.54	17.96	18.37	0.244	0.919
Jejunum + Ileum (%cm)	74.48	72.26	75.34	74.89	71.74	0.679	0.351

SEM: Standard error of the mean.

## Data Availability

The data presented in this study are available on request from the corresponding author.
